# Effects of the SARS-CoV-2 pandemic on women affected by endometriosis: a large cross-sectional online survey

**DOI:** 10.1080/07853890.2021.1991589

**Published:** 2021-10-29

**Authors:** Alessandro Arena, Benedetta Orsini, Eugenia Degli Esposti, Diego Raimondo, Jacopo Lenzi, Ludovica Verrelli, Raffaella Iodice, Paolo Casadio, Renato Seracchioli

**Affiliations:** a Gynecology and Human Reproduction Physiopathology, Dipartimento di Scienze Mediche e Chirurgiche (DIMEC), Via Massarenti, 13, IRCCS, Sant’Orsola-Malpighi Hospital, University of Bologna, Bologna, Italy; b Dipartimento di Scienze Biomediche e Neuromotorie (DIBINEM), IRCCS Azienda Ospedaliero-Universitaria di Bologna a, Bologna, Italy

**Keywords:** Anxiety, endometriosis, pandemic, post-traumatic stress disorder, SARS-CoV-2

## Abstract

**Introduction:**

The SARS-CoV-2 pandemic has forced healthcare providers to reorganize their activities to protect the population from infection, postponing or suspending many medical procedures. Patients affected by chronic conditions were among the most affected. In the case of catastrophes, women have a higher lifetime prevalence of post-traumatic stress disorder (PTSD), and those with endometriosis have higher anxiety levels, making them fragile in such circumstances.

**Materials and methods:**

In this cross-sectional study, conducted in May 2020, we considered all women aged ≥18 years, followed up at our referral centre for endometriosis. Patients were sent an anonymous 6-section questionnaire *via* email, containing different validated tools for the evaluation of anxiety levels and the risk of PTSD. A multivariable linear regression was performed to assess the impact of patients’ characteristics on the distress caused by the SARS-COV-2 pandemic.

**Results:**

Among the 468 women recruited, 68.8% were quite-to-extremely worried about not being able to access gynaecologic care, with almost one-third of them scoring ≥33 on the IES-R. Older age and increased levels of anxiety were associated with higher risks of PTSD (age: *b* = 0.28, 95% CI = 0.12 − 0.44; GAD-7: *b* = 1.71, 95% CI = 1.38 − 2.05), with up to 71.8% of patients with severe anxiety (GAD-7 > 15) having an IES-R score ≥33 suggestive for PTSD. Women who could leave home to work showed lower levels of PTSD (*b* = −4.79, 95% CI = −8.44 to − 1.15, ref. unemployed women). The implementation of telemedicine in routine clinical practice was favourably viewed by 75.6% of women.

**Discussion:**

Women with endometriosis are particularly exposed to the risk of PTSD during the SARS-CoV-2 pandemic, especially if they are older or have higher levels of anxiety. Gynaecologists should resort to additional strategies, and telemedicine could represent a feasible tool to help patients cope with this situation.

**KEY MESSAGES**
- The COVID-19 pandemic significantly impacted the lives of women with endometriosis, who appeared to have a considerable risk of PTSD.
- Older age, higher anxiety levels and unemployment were independently associated with the risk of developing PTSD.
- Clinicians should develop successful alternative strategies to help patients cope with this situation, and telemedicine might represent an applicable and acceptable solution.

## Introduction

On March 11th, 2020, the World Health Organization announced that viral pneumonia caused by SARS-CoV-2 could be characterized as a pandemic [1,2]. Healthcare providers were forced to revise their organization to protect patients and staff from the infection and to reallocate resources to answer to the acute needs of the Healthcare System. Moreover, the Italian government promptly reduced the activities that could be a source of contagion. The national authorities issued several restrictive regulations that strongly limited people’s circulation all over the Country, closing public utilities such as restaurants, bars, gyms and non-professional sport facilities, theatres and cinemas, shopping malls, and all non-grocery shops. Additionally, it was forbidden to move between different regions, and Italian people could not leave their houses unless they had an essential need to do so, like reaching their workplace or going to urgent medical appointments. These measures led to a forced lockdown, during which non-urgent medical activities were suspended and people were encouraged to leave their homes only for indispensable necessities. The dangers of additional waves of the pandemic have induced many countries to reinstate restrictive measures after adopting a more lenient attitude in the past months.

Routine outpatient evaluations for non-urgent conditions (i.e. oncological or life-threatening diseases) were among the suspended activities, mostly affecting patients suffering from chronic illnesses, such as endometriosis. Pain is one of its most striking characteristics, being the core of its symptomatology (dysmenorrhoea, ovulatory pain, dyspareunia, chronic pelvic pain, dyschezia, dysuria) and significantly worsening women’s quality of life [3,4]. As previously demonstrated, endometriosis takes a hard toll on women’s psychological wellbeing, in particular increasing their anxiety levels [5,6]. Indeed, the management of obstetric and gynecological patients has been negatively affected by the COVID-19 pandemic, with non-negligible effects especially on pregnant women [7–10].

Although great care has been taken in order to identify, isolate, and treat people affected by SARS-CoV-2, the management of the psychological impact of this pandemic has been partially neglected [11]. Catastrophic disasters can cause overwhelming physical and mental distress, and this seems especially true for women, who have a higher lifetime prevalence of post-traumatic stress disorder (PTSD) [12]. Social distancing and quarantine – imposed to reduce viral transmission – increased anxiety and feelings of isolation, as demonstrated by several studies conducted during the 2003-SARS outbreak [13]. Even more, information on experiences of SARS-CoV-2 in the general population shows more anxiety and depression compared to historical norms [14]. In light of this, women with endometriosis may have a greater risk of developing higher distress levels than the general population, and the psychological impact of this pandemic on them should be investigated. Our study aims to assess the relationship between patients’ demographic and clinical characteristics and the results of the Impact of Event Scale-Revised (IES-R) questionnaire in a cohort of women affected by endometriosis followed-up at our academic referral centre. Moreover, we analysed the relationship between the intensity of endometriosis-related symptoms and anxiety levels and the psychological impact of the SARS-CoV-2 pandemic on these women.

## Materials and methods

This cross-sectional study was conducted after the lockdown phase, from May 4th to May 31st, 2020, at our academic centre for endometriosis and pelvic pain, in compliance with the STROBE guidelines. The study protocol was approved by the local Ethics Committee (201/2017/O/Sper).

All women aged 18 years or more who had previously been evaluated at our institution and who had received a clinical and sonographic diagnosis of endometriosis were considered for this study. These patients were contacted *via* phone-call to explain the purposes of this research and to acquire their informed consent to participate. Patients who agreed were then sent an anonymous 6-section questionnaire, created with a private Google form, *via* email. In order to begin to fill it in, women had to give their informed consent again, both regarding the participation to the study and the processing of personal data, after carefully reading specific informative forms.

The first section contained anamnestic and demographic information, which is normally acquired during patients’ examination at our medical centre, as per our usual clinical practice. We included in this section also the evaluation of exclusion criteria (ongoing pregnancy, postmenopausal status, history of malignancies, previous diagnosis of psychiatric diseases according to the DSM-V [15] and/or current therapy with psychotropic drugs). Women residing outside of Italy during the lockdown were also ruled out from the analysis, to avoid biases owing to the different rules imposed by foreign Governments. Information regarding patients’ age, marital status, level of education, gravidity and parity, and previous history of endometriosis was acquired.

Based on feedbacks and concerns previously voiced by some of our patients, we elaborated additional questions to investigate which everyday life facets affected women’s distress the most during the COVID-19 pandemic. Thus, women’s working habits during the pandemic, their concerns about obtaining drugs prescriptions and outpatient gynecological appointments during the lockdown period were investigated, as well as their attitudes towards telemedicine [16]. In particular, participants were asked how favourable they would be to the implementation of telemedicine in our routine practice (ranking their preference from 0 to 5, 0 = not favourable at all, 5 = extremely favourable). The severity of endometriosis-related symptoms, assessed through the Numerical Rating Scale (NRS), was also measured, along with patients’ levels of sexual satisfaction during the lockdown phase.

The second section contained the Italian version of the dedicated 11-item questionnaire of Endometriosis Health Profile 5 (EHP-5) [17]. Anxiety levels were then estimated in the third and fourth sections, using the Italian version of Generalized Anxiety Disorder −7 (GAD-7) and the Spielberg State Trait Anxiety Inventory Y6 (STAI-Y6) [18,19]. The STAI Y6 enables patients to rate the degree to which they are currently experiencing anxiety using a 4-point scale that ranges from 1 (not at all) to 4 (very much so). The total scores are recorded from 20 to 80 according to the common usage. The GAD-7 is a valid standardized self-report tool used to screen and assess the severity of generalized anxiety. All of these questionnaires are normally submitted to our patients during medical consultations at our clinic, since their validity to assess the psychological impact of endometriosis on women’s life has amply been investigated [5].

In the last section, the psychological impact of the SARS-CoV-2 pandemic was appraised through the Italian version of Impact of Event Scale-Revised (IES-R) [20], which is a useful tool to appraise the risk of post-traumatic stress disorder (PTSD) and which has already been used to investigate the effects of this pandemic in other settings [21,22]. Women were asked to rate how strongly their lives were affected by the COVID-19-induced lockdown period and how many difficulties they encountered during their everyday life during that period. The IES-R is composed of 22 items and includes 3 subscales: intrusion, avoidance, and hyperarousal. Patients have to rate each item on a scale from 0 (i.e. not at all) to 4 (i.e. extremely). The total score varies between 0 and 88 and is generally divided into 4 subgroups from normal to severe psychological impact. Scores higher than 24 indicate a relevant concern for PTSD, although many people with scores this high may only have partial forms of PTSD or present with some of the symptoms. According to the available literature, we used a score of 33 as the best cut-off for a probable diagnosis of PTSD [20,23].

All these tools and scales have been validated before by several studies and are fully and easily accessible.

### Statistical analysis

Numerical variables were summarized as mean ± standard deviation; categorical variables were summarized as frequencies and percentages. A multivariable linear regression was performed to study the impact of demographic and clinical characteristics on the distress caused by the SARS-CoV-2 pandemic, as measured by the Impact of Event Scale (IES-R) total score. Due to the descriptive nature of the analysis, we did not perform any automated selection of the variables to be included in the multivariable regression model. Normality of residuals was checked with the normal Q–Q plot and the Shapiro–Wilk test; correct model specification was confirmed with the Ramsey regression specification-error test for omitted variables; linearity of numerical predictors was verified *via* augmented component-plus-residual plots; no multi-collinearity issues were found. Huber–White standard errors were used to deal with the presence of heteroscedastic residuals. In a secondary analysis, the single items of EHP-5, GAD-7, and STAI-Y6 were included in the model as potential predictors of post-traumatic distress.

All analyses were carried out using Stata software, version 15 (StataCorp, 2017, *Stata Statistical Software: Release 15,* College Station, Texas, USA: StataCorp LP). The significance level was set at 5%.

## Results

During the study period, a total of 1226 women were contacted *via* phone in order to ask them to participate in this study. Two hundred and fifteen patients refused their consent and were not further contacted. The remaining 1011 received our questionnaire *via* email: 305 of them failed to respond to it and 175 of them did not complete all the sections. Two women were living abroad during the lockdown phase and were therefore excluded. Finally, 61 patients were ruled out based on the other exclusion criteria.

**Figure 1.**
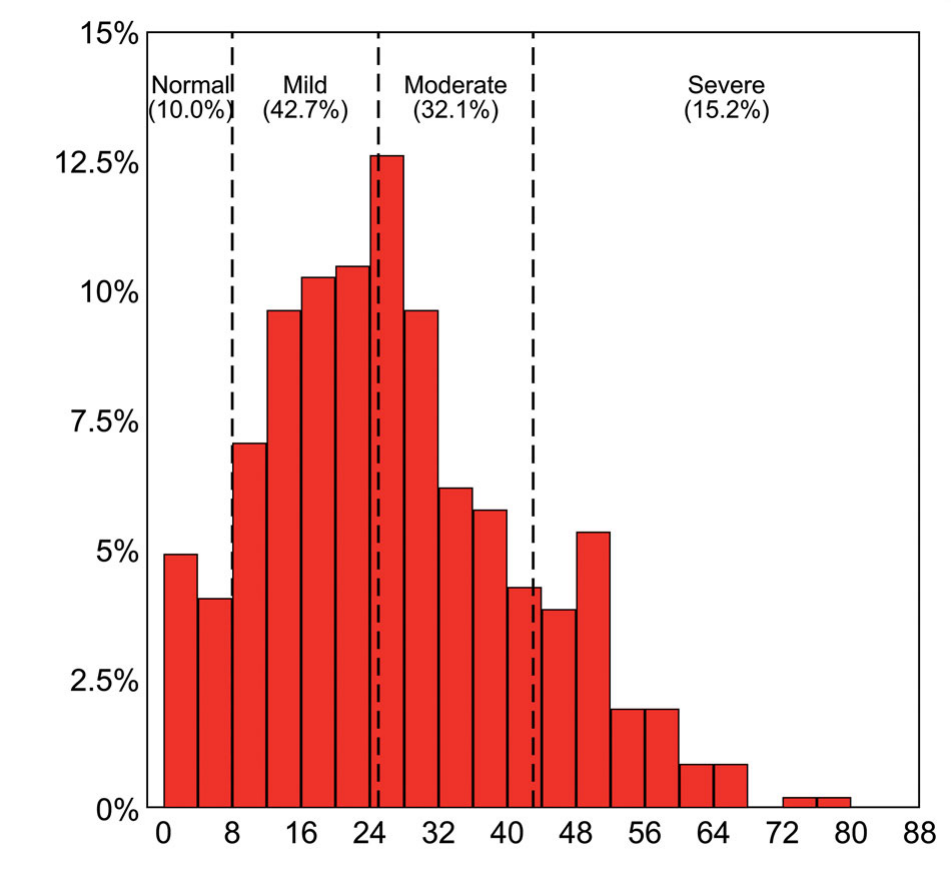
Percentage distribution of Impact of Event Scale (IES-R) total score among the study participants (*n* = 468). *Notes:* IES-R cut-offs to define normal (0–8), mild (9–25), moderate (26–43) and severe (44–88) post-traumatic stress symptoms are marked as dashed vertical lines.

The characteristics of the remaining 468 patients are summarized in Table 1. The mean age was 38.8 ± 7.7 years. Most patients worked from home during the lockdown phase (37.8%). As detailed in Table 1, most women (68.6%) were quite to extremely worried about not having access to gynaecologic care during the pandemic, and 63.5% of them were quite-to-extremely concerned for their health. The distribution of the IES-R total score is illustrated in Figure 1. Mean score was estimated to be 26.4 ± 15.0 (interquartile range = 15–36); 140 patients (29.9%) had a total score ≥33, which indicates a probable diagnosis of PTSD.

**Table 1.** Characteristics of the study patients (*n* = 468). Values are counts (percentages) or mean ± standard deviation [interquartile range].

Patient characteristic	
Age, y	38.8 ± 7.7 [33–45]
Age group, y	
≤20	3 (0.6)
21–29	49 (10.5)
30–39	190 (40.6)
40–49	192 (41.0)
≥50	34 (7.3)
Living alone	66 (14.1)
Being a parent	151 (32.3)
Educational attainment	
Lower secondary	36 (7.7)
Upper secondary	196 (41.9)
Tertiary	176 (37.6)
Other	60 (12.8)
Work during lockdown	
Unemployed	70 (15.0)
Stopped working	87 (18.6)
Worked from home	177 (37.8)
Went to work	134 (28.6)
Previous surgery for endometriosis	300 (64.1)
Waitlisted for surgery	26 (5.6)
Seeking pregnancy before lockdown	
No	386 (82.5)
Yes, not postponed	54 (11.5)
Yes, postponed	28 (6.0)
Waitlisted for fertility testing	
No	429 (91.7)
Yes, postponed by the hospital	26 (5.6)
Yes, postponed by me	13 (2.8)
Hormonal treatment during lockdown	
No	159 (34.0)
Yes, easy to access	241 (51.5)
Yes, a little difficult to access	26 (5.6)
Yes, quite difficult to access	24 (5.1)
Yes, very difficult to access	11 (2.4)
Yes, virtually impossible to access	7 (1.5)
Concerned about not having access to specialist care	
No	58 (12.4)
A little	89 (19.0)
Quite	153 (32.7)
Very	91 (19.4)
Extremely	77 (16.5)
Worried for her health	
No	99 (21.2)
A little	72 (15.4)
Quite	160 (34.2)
Very	101 (21.6)
Extremely	36 (7.7)
Dysmenorrhoea, NRS	2.5 ± 3.2 [0–5]
Presence of dysmenorrhoea (NR*S* > 0)	233 (49.8)
Chronic pelvic pain, NRS	2.8 ± 3.0 [0–5]
Presence of chronic pelvic pain (NR*S* > 0)	282 (60.3)
Dyspareunia, NRS	2.4 ± 3.2 [0–4]
Presence of dyspareunia (NR*S* > 0)	227 (48.5)
Dyschezia, NRS	2.1 ± 2.8 [0–4]
Presence of dyschezia (NR*S* > 0)	239 (51.1)
Dysuria, NRS	1.1 ± 2.2 [0–1]
Presence of dysuria (NR*S* > 0)	129 (27.6)
Worsening of symptoms during lockdown	61 (13.0)
Sexual intercourses during lockdown	274 (58.5)
EHP-5 scoring	25.4 ± 20.2 [11.4–36.4]
GAD-7 scoring	7.9 ± 4.8 [4–11]
STAI-Y6 scoring	48.4 ± 13.2 [36.7–60.0]

NRS: numeric rating scale; EHP-5: Short Form Endometriosis Health Profile; GAD-7: Generalized Anxiety Disorder 7-item; STAI-Y6: Spielberg State Trait Anxiety Inventory 6-item.

Results of simple (bivariate) regression analysis are presented in Table 2. On multivariable analysis (Table 3), we found that older age and increased level of generalized anxiety were independently associated with higher post-traumatic distress (age: *b* = 0.28, 95% CI = 0.12 − 0.44; GAD-7: *b* = 1.71, 95% CI = 1.38 − 2.05). As further proof of this result, we found that 59.5% of patients who had moderate generalized anxiety levels (GAD-7 score 11–15) and 71.8% of those with severe anxiety (GAD-7 score >15) had an IES-R score ≥33, as compared to 6.1% and 28.6% of women who had no (GAD-7 score ≤5) or mild anxiety (GAD-7 score 6–10), respectively (*p* < .001). Based on the GAD-7 scores, 56.8% of the patients could be diagnosed with a mild-to-moderate form of generalized anxiety.

**Table 2.** Results of simple (bivariate) linear regression analysis for subjective distress caused by SARS-COV-2 pandemic, as measured by the Impact of Event Scale (IES-R) total score.

Patient characteristic	Regression coefficient	*p*-value	95% confidence interval	
			Lower bound	Upper bound
Age, y	0.16	.080	–0.02	0.35
Living alone				
No	Ref.			
Yes	3.05	.149	–1.10	7.20
Being a parent				
No	Ref.			
Yes	–1.16	.442	–4.14	1.81
Educational attainment				
Lower secondary	Ref.			
Upper secondary	1.77	.517	–3.60	7.15
Tertiary	0.73	.788	–4.57	6.02
Other	1.31	.680	–4.91	7.53
Work during lockdown				
Unemployed	Ref.			
Stopped working	–3.63	.127	–8.30	1.04
Worked from home	–3.82	.094	–8.30	0.66
Went to work	–7.94	.001*	–12.41	–3.46
Previous surgery for endometriosis				
No	Ref.			
Yes	0.40	.779	–2.42	3.23
Waitlisted for surgery				
No	Ref.			
Yes	1.51	.583	–3.90	6.92
Seeking pregnancy before lockdown				
No	Ref.			
Yes, not postponed	–2.85	.155	–6.78	1.08
Yes, postponed	–2.08	.496	–8.09	3.92
Waitlisted for fertility testing				
No	Ref.			
Yes, postponed by the hospital	–0.17	.947	–5.20	4.86
Yes, postponed by me	–4.67	.189	–11.65	2.31
Hormonal treatment during lockdown				
No	Ref.			
Yes, easy to access	–0.19	.902	–3.14	2.77
Yes, a little difficult to access	0.09	.979	–6.56	6.74
Yes, quite difficult to access	3.08	.305	–2.82	8.99
Yes, very difficult/virtually impossible to access	11.35	.007*	3.13	19.57
Concerned about not having access to specialist care				
No	Ref.			
A little	1.02	.650	–3.39	5.42
Quite	5.09	.014*	1.04	9.14
Very	3.84	.084	–0.51	8.18
Extremely	12.46	<.001*	7.67	17.24
Worried for her health				
No	Ref.			
A little	4.04	.066	–0.27	8.35
Quite	5.71	.001*	2.36	9.06
Very	11.42	<.001*	7.25	15.59
Extremely	14.14	<.001*	7.23	21.04
Dysmenorrhoea, NRS	0.83	<.001*	0.38	1.27
Chronic pelvic pain, NRS	1.24	<.001*	0.78	1.69
Dyspareunia, NRS	1.05	<.001*	0.62	1.48
Dyschezia, NRS	1.32	<.001*	0.83	1.81
Dysuria, NRS	1.16	<.001*	0.56	1.76
Worsening of symptoms during lockdown				
No	Ref.			
Yes	6.13	.005*	1.83	10.42
Sexual intercourses during lockdown				
No	Ref.			
Yes	–2.10	.138	–4.88	0.68
EHP-5 scoring	0.04	.276	–0.03	0.10
GAD-7 scoring	1.98	<.001*	1.74	2.21
STAI-Y6 scoring	0.54	<.001*	0.45	0.63

*Significant at the 5% level.

**Table 3.** Results of multivariable linear regression analysis for subjective distress caused by SARS-COV-2 pandemic, as measured by the Impact of Event Scale (IES-R) total score.

Patient characteristic	Regression coefficient	*p*-value	95% confidence interval	
			Lower bound	Upper bound
Age, y	0.28	.001*	0.12	0.44
Living alone				
No	Ref.			
Yes	1.66	.371	–1.99	5.32
Being a parent				
No	Ref.			
Yes	0.41	.710	–1.77	2.60
Educational attainment				
Lower secondary	Ref.			
Upper secondary	1.62	.531	–3.46	6.70
Tertiary	2.62	.309	–2.43	7.66
Other	4.40	.126	–1.25	10.04
Work during lockdown				
Unemployed	Ref.			
Stopped working	–1.99	.329	–6.00	2.01
Worked from home	–3.31	.069	–6.88	0.26
Went to work	–4.79	.010*	–8.44	–1.15
Previous surgery for endometriosis				
No	Ref.			
Yes	0.28	.820	–2.13	2.69
Waitlisted for surgery				
No	Ref.			
Yes	–2.27	.362	–7.15	2.62
Seeking pregnancy before lockdown				
No	Ref.			
Yes, not postponed	1.11	.528	–2.34	4.56
Yes, postponed	0.58	.844	–5.23	6.39
Waitlisted for fertility testing				
No	Ref.			
Yes, postponed by the hospital	–0.42	.871	–5.55	4.71
Yes, postponed by me	–2.98	.295	–8.56	2.60
Hormonal treatment during lockdown				
No	Ref.			
Yes, easy to access	1.98	.127	–0.57	4.53
Yes, a little difficult to access	0.13	.954	–4.42	4.69
Yes, quite difficult to access	4.62	.071	–0.40	9.64
Yes, very difficult/virtually impossible to access	6.87	.020*	1.11	12.63
Concerned about not having access to specialist care				
No	Ref.			
A little	–1.03	.571	–4.58	2.53
Quite	1.46	.424	–2.13	5.05
Very	–0.62	.750	–4.42	3.19
Extremely	2.28	.303	–2.06	6.62
Worried for her health				
No	Ref.			
A little	3.15	.082	–0.40	6.70
Quite	3.64	.014*	0.74	6.55
Very	5.42	.003*	1.85	8.99
Extremely	5.48	.062	–0.27	11.23
Dysmenorrhoea, NRS	0.28	.224	–0.17	0.74
Chronic pelvic pain, NRS	0.10	.729	–0.49	0.70
Dyspareunia, NRS	0.39	.071	–0.03	0.81
Dyschezia, NRS	0.34	.191	–0.17	0.84
Dysuria, NRS	–0.55	.062	–1.13	0.03
Worsening of symptoms during lockdown				
No	Ref.			
Yes	0.32	.862	–3.26	3.90
Sexual intercourses during lockdown				
No	Ref.			
Yes	–0.02	.985	–2.30	2.26
EHP-5 scoring	–0.01	.748	–0.06	0.04
GAD-7 scoring	1.71	<.001*	1.38	2.05
STAI-Y6 scoring	0.01	.796	–0.10	0.13

The adjusted *R^2^* of the model is 0.432. * Significant at the 5% level.

Patients who found it difficult to obtain hormonal therapy during the lockdown had increased distress levels as compared to those who were not under treatment (*b* = 6.87, 95% CI = 1.11 − 12.63, ref. women without hormonal therapy); a similar result was found among women who were worried about their health status as a result of the SARS-CoV-2 pandemic. On the contrary, those who left their houses to go to work during the lockdown period had lower levels of post-traumatic distress, as compared to unemployed women (*b* = −4.79, 95% CI = −8.44 to −1.15, ref. unemployed women).

Some items of the GAD-7 were also significantly associated with the study outcome (Table 4).

**Table 4.** Relationship of specific signs of generalized anxiety disorder (GAD-7 items) with subjective distress caused by SARS-COV-2 pandemic, as measured by the Impact of Event Scale (IES-R) total score.

Items of the GAD-7 scale	Regression coefficient	*p*-value	95% confidence interval	
			Lower bound	Upper bound
Feeling nervous, anxious, or on edge	1.22	.340	–1.29	3.73
Not being able to stop or control worrying	–0.27	.828	–2.72	2.18
Worrying too much about different things	3.32	.009*	0.82	5.82
Trouble relaxing	1.19	.265	–0.91	3.28
Being so restless that it is hard to sit still	2.11	.035*	0.15	4.07
Becoming easily annoyed or irritable	1.77	.044*	0.05	3.49
Feeling afraid as if something awful might happen	2.87	.002*	1.02	4.72

Regression coefficients are adjusted for the demographic and clinical characteristics listed in Tables 1 and 2. *Significant at the 5% level.

Regarding patients’ perception of telemedicine, 75.6% declared themselves well-disposed towards it (moderately favourable 24.1%, favourable 19.2%, extremely favourable 32.3%).

## Discussion

The SARS-CoV-2 pandemic showed a high psychological impact on women with endometriosis, who have a significant risk of PTSD. In particular, the risk seemed higher for women already suffering from mild-to-moderate generalized anxiety, older women, and unemployed ones. Also, women who could not easily have access to medical therapy experienced higher levels of distress. No association was found between the IES-R score and the EHP-5 or the STAI-Y6 scores.

Our results show an interesting correlation between the GAD-7 results and the IES-R score. This is not surprising, demonstrating that women who had higher anxiety levels before the pandemic scored higher on the IES-R questionnaire, having a greater risk of developing PTSD afterward. Indeed, we found a mean GAD-7 scoring of 7.9 ± 4.8, indicating that most women in our cohort could be diagnosed with a mild-to-moderate form of generalized anxiety disorder. Naturally, the partial overlap existing between the symptoms of generalized anxiety and PTSD must be taken into account to explain in part this association. However, we deemed it quite interesting that the percentage of women who scored ≥ 33 on the IES-R questionnaire increased with the severity of their generalized anxiety: almost 3 out of 4 patients in the “severe” GAD-7 category turned out to be at risk for developing PTSD. Furthermore, the GAD-7 questionnaire is itself a valid tool to diagnose PTSD, with high accuracy at a cut-point of ≥8, which is very similar to our results, further underlying the reliability of the correlation we found [24].

Unexpectedly, no significant correlation was found between the results of the EHP-5 and the STAI-Y6 and the IES-R scores, nor did women report worsened symptoms during the lockdown. Indeed, the relationship between pain symptoms and PTSD is controversial and other authors did not report a significant impact of COVID-19 in endometriosis’ symptoms as well [25]. After the Great East Japan Earthquake, the relationship between dysmenorrhoea in Japanese adolescents was investigated, calling into question the higher levels of the cytokines TNF-α and IL-1 detected in PTSD patients, which could enhance the production of pain mediators [26]. On the other hand, Seng et al. did not found any significant associations between the simple-PTSD category and dysmenorrhoea or chronic pelvic pain [27]. As for the STAI-Y6, we found no significant correlation with the IES-R scoring, as opposed to the GAD-7. The first questionnaire evaluates state anxiety (i.e. anxiety about a specific event), and not only trait anxiety, as a personal characteristic [18,19]. Since our patients were contacted immediately after the lockdown phase, they probably answered the questionnaires in a comfortable setting, so it is possible that the STAI-Y6 might be lower than expected.

No correlation was demonstrated between the IES-R score and having delayed pregnancy or fertility treatments. The majority of women who had been seeking pregnancy before the SARS-CoV-2 outbreak did not actually stop trying for a baby as compared to only 28 who decided to wait (Table 1), either on their own or due to the cancellation of fertility treatments. The lack of an evincible association may thus depend on the small number of women considered. Some authors found that fertility treatment cancellation led to high levels of anxiety and distress, among both women and men [28].

The difficulty or impossibility to obtain medical therapy during the lockdown phase, although temporary, appeared as one of the main concerns of our patients, influencing the IES-R. Medical therapy is essential in the management of endometriosis, reducing and controlling painful symptoms, although complete surgical eradication is sometimes necessary to restore the normal pelvic anatomy and re-establish pelvic organ functions[29–32]. The majority of women in this study had already been submitted to surgical excision of endometriosis, and they were conscious of the importance of maintaining a long-term medical therapy to avoid the risk of symptoms recurrence at drug discontinuation [33].

Isolation seems to play a decisive role, too: women who had the opportunity to leave home to go to work had lower IES-R scores by 4.79 points on average. This result may seem extraordinary, given the higher danger these women have of becoming infected. However, forced quarantine was found to be associated with negative responses in patients, such as fear, sadness, anxiety-induced insomnia, and anger. Additionally, house-confinement and working from home worsen behavioural changes in terms of diet, sleep, and even addiction, increasing SARS-CoV-2-linked anxiety and vice-versa [34,35]. Also, greater job insecurity due to SARS-CoV-2 was associated with worse anxiety symptoms [36]. Women who retained the possibility to work might have felt more secure, partially explaining our results. It should also be noted that endometriosis per se severely impacts many women’s working lives, forcing them to miss several workdays when symptoms are at their worse and reducing their productivity [37], further explaining our results.

Older people scored higher in the IES-R questionnaire, with the score increasing on average by 0.28 points for every year of age. This finding is in contrast to what is reported in the literature. Various authors found a higher prevalence of depression, anxiety, and PTSD in younger people compared to people over 40 years of age [35,38]. Based on our experience, younger women have a more recent diagnosis of endometriosis and are less likely to have suffered from painful symptoms for a long time, compared to older patients, who had had to live with endometriosis and its drastic consequences on their quality of life for years.

Finally, we found a relatively high incidence of PTSD among endometriotic women, compared to other data available in the literature regarding the general population [35,38]. In the Spanish experience, the incidence of PTSD symptoms was 15.8%, whereas the Chinese report an incidence of 12.8%, significantly lower than our 29.9%. However, both studies used the Civilian version of the Post-traumatic Stress Disorder Checklist-reduced version questionnaire to evaluate the presence of PTSD, making it difficult to draw definitive conclusions. Endometriosis is accompanied by a deterioration of quality of life, owing to chronic pain, the necessity of chronic therapy and often invasive surgery, and concerns about fertility [28]. Furthermore, during the SARS-CoV-2 outbreak, our patients were faced with the temporary impossibility to reach for a consult or an outpatient evaluation and this could have been a contributing factor to the higher risk of PTSD.

The SARS-CoV-2 pandemic represents one of the most worrisome catastrophes of the last decades, changing people’s everyday lives and forcing world healthcare systems to reorganize their activities. In this scenario, people suffering from chronic benign conditions, such as endometriosis, were obliged to forgo their regular outpatient evaluations during the most restrictive lockdown phase, with the risk of feeling neglected and isolated.

In this new scenario, alternative ways to follow-up chronic patients should be searched, providing them with continual support and advice while adhering to the new restrictions. The implementation of telemedicine has rapidly become one of the most useful tools in clinical settings. Triggered by the SARS-CoV-2 pandemic, the volume of telemedicine dramatically increased, enabling healthcare providers to restore a significant proportion of their typical clinic volume and preventing a massive disruption to the routine patient-care workflow. Interestingly, the majority of our patients declared themselves favourable to its use, with 32.3% being extremely favourable to it, both as a preliminary screening method before an in-patient visit and as a substitute for it, in agreement with what is reported in the literature [25].

Given these results, it could be useful to evaluate patients’ anxiety levels with the GAD-7 questionnaire before routine outpatient evaluations. Thus, gynaecologists might have an insight into each woman’s psychological background, improving the doctor-patient relationship, and to create an effective therapeutic relationship. Additionally, the implications of telemedicine and its cost-effectiveness in a gynecological setting should be further investigated, to better define its role, advantages, and possible limitations.

To the best of our knowledge, this survey is the first study examining the psychological consequences of the SARS-CoV-2 pandemic on women affected by endometriosis, despite presenting some limitations. First of all, this is a single-centre experience,reflecting only the effects of this pandemic on a part of the population living in Italy. Anyway, most governments all over the world were forced to adopt similar strategies to contain the pandemic, making our results quite reliable. Moreover, patients filled in this questionnaire immediately after the lockdown phase, thus a certain recall bias must be taken into account. Additionally, our questionnaires were administered through an online form and filled in at home. On the other hand, the large study population represents its greater strength, increasing our awareness that women with endometriosis could represent a particularly fragile category of patients that should not be neglected in times of stressful global events. However, as no control group was enrolled in our study and only cross-sectional data were collected, care should be taken before drawing definite conclusions. Further studies would help clarify this issue, also analysing the incidence and prevalence of depressive symptoms, which are frequent among women suffering from endometriosis [39].

The SARS-CoV-2 pandemic has determined a high psychological impact on endometriotic women, who have a significant risk of PTSD. In particular, risks seemed higher for women already suffering from mild-to-moderate generalized anxiety, older women, and unemployed ones. Since the end of the pandemic is far at present, these findings should prompt clinicians to develop successful strategies to help patients cope with this difficult moment, especially those suffering from chronic diseases. The implementation of telemedicine in the routine clinical practice, in the form of video-conferences or phone consultations, might represent an applicable and convenient alternative, allowing gynaecologists to reduce women’s feeling of isolation and need.

## Data Availability

Tha data analysed in this paper cannot be made open in compliance with the study protocol.
